# High body mass index is associated with elevated risk of perioperative ischemic stroke in patients who underwent noncardiac surgery: A retrospective cohort study

**DOI:** 10.1111/cns.14838

**Published:** 2024-07-10

**Authors:** Peng Li, Rui Wang, Fengjin Liu, Libin Ma, Huikai Yang, Mengyao Qu, Siyuan Liu, Miao Sun, Min Liu, Yulong Ma, Weidong Mi

**Affiliations:** ^1^ Department of Anesthesiology The Sixth Medical Center of Chinese PLA General Hospital Beijing China; ^2^ Department of Anesthesiology The First Medical Center of Chinese PLA General Hospital Beijing China; ^3^ Nation Clinical Research Center for Geriatric Diseases Chinese PLA General Hospital Beijing China; ^4^ Department of Emergency Yantai Yuhuangding Hospital Shandong China; ^5^ Department of Anesthesiology, Beijing Tongren Hospital Capital Medical University Beijing China

**Keywords:** BMI, continuous variable, non‐cardiovascular surgery, optimal cutoff value, perioperative ischemic stroke

## Abstract

**Background:**

Body mass index (BMI) serves as a global metric for assessing obesity and overall health status. However, the impact of BMI, treated as a continuous variable, on the risk of perioperative stroke remains poorly understood. This retrospective cohort study aimed to elucidate the association between BMI and the risk of perioperative ischemic stroke in patients undergoing non‐cardiovascular surgery.

**Methods:**

A cohort of 223,415 patients undergoing noncardiac surgery at the First Medical Center of Chinese PLA General Hospital between January 1, 2008 and August 31, 2019 was screened. Preoperative high BMI, defined as BMI >22.64 kg/m^2^, was the primary exposure, and the outcome of interest was the new diagnosis of perioperative ischemic stroke within 30 days post‐surgery. Robust controls for patient and intraoperative factors were implemented to minimize residual confounding. Logistic regression and propensity score matching were employed, and patients were stratified into subgroups for further investigation.

**Results:**

The overall incidence of perioperative ischemic stroke was 0.23% (*n* = 525) in the cohort. After adjusting for patient‐related variables (OR 1.283; 95% CI, 1.04–1.594; *p* < 0.05), surgery‐related variables (OR 1.484; 95% CI, 1.2–1.849; *p* < 0.001), and all confounding variables (OR 1.279; 95% CI, 1.025–1.607; *p* < 0.05), patients with BMI >22.64 kg/m^2^ exhibited a significantly increased risk of perioperative ischemic stroke. This association persisted in the propensity score matched cohort (OR 1.577; 95% CI, 1.203–2.073; *p* < 0.01). Subgroup analyses indicated that preoperative BMI >22.64 kg/m^2^ correlated with an elevated risk of perioperative ischemic stroke in female patients, those with coronary heart disease, peripheral vascular diseases, and individuals undergoing neurosurgery.

**Conclusion:**

We first identified BMI >22.64 kg/m^2^ as a substantial and independent risk factor for perioperative ischemic stroke in Chinese noncardiac surgery patients. Normal BMI may not suffice as a universal preventive standard. Instead, a more stringent perioperative weight management approach is recommended, particularly for specific subgroups such as female patients, those with coronary heart disease and peripheral vascular disease, and individuals scheduled for neurosurgery.

## Graphical abstract

BMI >22.64 kg/m^2^ emerges as a substantial and independent risk factor for perioperative ischemic stroke. A more stringent perioperative weight management approach is recommended, particularly for specific subgroups such as female patients, those with coronary heart disease and peripheral vascular disease, and individuals scheduled for neurosurgery.

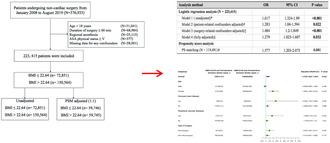

## 1 INTRODUCTION

Stroke stands as the world's second leading cause of death, contributing to 10%–15% of global mortality.^1^ Perioperative stroke, defined as ischemic or hemorrhagic cerebral infarction during or within 30 days after surgery, though occurring at a relatively low incidence (0.1–1.0%),^2^, ^3^, ^4^ carries a staggering fatality rate of approximately 50% within a decade.^5^ Hindered by delayed diagnostic imaging, a narrow intervention window, and heightened bleeding risks, <5% of eligible patients benefit from thrombolysis, resulting in a majority facing a bleak prognosis.^6^ Recognizing the urgency of this issue, active preoperative intervention in risk factors emerges as pivotal for averting perioperative stroke occurrences.

Body mass index (BMI), derived by dividing weight (kg) by the square of height (m), serves as an international standard gauging obesity and overall health. Elevated BMI often leads to metabolic disorders, predisposing individuals to various diseases. Studies indicate that a BMI of ≥30 kg/m^2^ is a risk factor for conditions such as hypertension, diabetes, heart failure, ischemic heart disease, and stroke.^7^, ^8^, ^9^, ^10^ In the realm of surgical patients, a BMI of ≥30 kg/m^2^ significantly escalates the risks of postoperative infection, pulmonary complications, and postoperative mortality.^11^, ^12^, ^13^ However, the relationship between BMI and perioperative stroke remains contentious.

Previous investigations have yielded conflicting findings; a retrospective study identified a BMI of ≥25 kg/m^2^ as an independent risk factor for perioperative stroke in patients undergoing percutaneous coronary intervention.^14^ Conversely, two large‐scale studies proposed that a higher BMI (35.0–40.0 kg/m^2^) may confer protective effects against perioperative stroke.^3^, ^15^ Meanwhile, several clinical studies found no significant association between BMI and perioperative stroke risk.^8^, ^16^ Notably, these studies treated BMI as a categorical variable, despite its inherent nature as a continuous measure reflecting the nuanced relationship between weight and height.

To address this gap, we present the first systematic study exploring the association between BMI as a continuous variable and the risk of perioperative ischemic stroke. This retrospective study encompasses 223,415 Chinese noncardiac surgery patients and aims to shed light on the nuanced relationship between BMI and perioperative stroke risk. Given the ethnic variations in BMI classifications and the absence of research on this topic within the Chinese surgical population, our study provides valuable insights to the global understanding of perioperative stroke risks.

## 2 METHODS

### 2.1 Ethical approval and compliance

The study was conducted in accordance with the approved research protocol by the Medical Ethics Committee of the Chinese PLA General Hospital (reference number: S2021‐493‐01). The requirement for written informed consent was waived. The manuscript adheres to the applicable Strengthening the Reporting of Observational Studies in Epidemiology (STROBE) guidelines (Table S1).

### 2.2 Study design and participant selection

**FIGURE 1:**
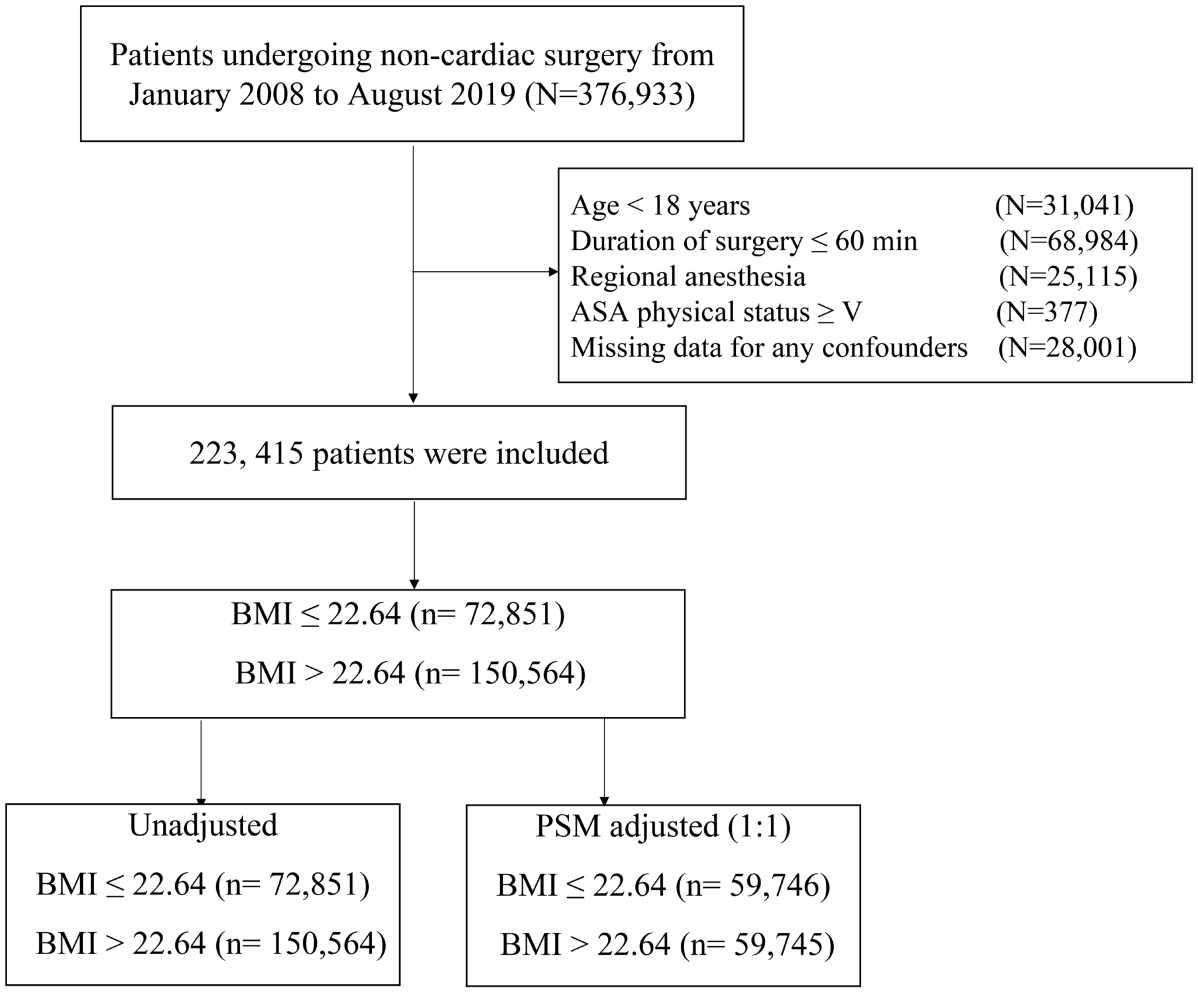
Study flow diagram. ASA, American Society of Anesthesiologists; BMI, body mass index; PSM, propensity score matching.

This retrospective cohort study included patients who underwent noncardiac surgery at the First Medical Center of Chinese PLA General Hospital, a tertiary referral academic hospital in Beijing, China, between January 1, 2008 and August 31, 2019. Patients meeting the following criteria were included: (1) undergoing noncardiac surgery, (2) age ≥ 18 years, (3) duration of surgery >60 min, (4) general anesthesia, (5) American Society of Anesthesiologists (ASA) physical status <V, and (6) complete data for all confounders. For individuals with multiple surgeries during the study period, only data from the first qualifying surgery were included. Patients diagnosed with perioperative ischemic stroke were identified through ICD‐9/10 diagnosis codes (Table S2). A flowchart illustrating the patient screening process is presented in Figure 1.

### 2.3 Outcome and exposure measures

The primary outcome of interest was perioperative ischemic stroke, defined as neurological dysfunction (motor, sensory, or cognitive) attributable to focal cerebral, spinal, or retinal infarction within 30 perioperative days.^17^ Diagnosis was confirmed through discharge records containing at least one ICD‐9‐CM/ICD‐10‐CM code for stroke (Table S2). The exposure of interest was preoperative body mass index (BMI), which was stratified into BMI ≤22.64 kg/m^2^ and BMI >22.64 kg/m^2^ based on receiver operating characteristic curve analysis.

### 2.4 Covariates and data collection

We considered 34 potential confounders, categorized as patient‐related and surgery‐related. Patient‐related confounders included: age, sex, ASA classification, hypertension, diabetes mellitus, stroke, history of chronic cerebrovascular disease, coronary heart disease, arterial fibrillation, valvular heart disease, myocardial infarction, history of cardiac surgery, peripheral vascular disease, renal dysfunction, preoperative use of β‐blockers, aspirin, and statin; as well as indices derived from the preoperative laboratory data preoperative, such as hemoglobin, albumin, total bilirubin, prothrombin time, neutrophil‐to‐lymphocyte ratio (NLR), and platelet‐to‐lymphocyte ratio (PLR). These were collected from most recent blood counts measured within 3 days prior to surgery. Surgery‐related confounders encompassed surgical type, duration, time of perioperative mean arterial pressure (MAP) >60 min, estimated blood loss, blood products depot, morphine equivalents, inhalation anesthetics, hormones, nonsteroidal anti‐inflammatory drugs (NSAIDs), colloids infusion, and crystalloids infusion.

### 2.5 Statistical analysis

Logistic regression analysis was employed to assess the relationship between BMI and the risk of perioperative ischemic stroke. Four models were constructed: Model 1 (unadjusted), Model 2 (adjusted for patient‐related confounders), Model 3 (adjusted for surgery‐related confounders), and Model 4 (fully adjusted to patient‐related and surgery‐related confounders). To enhance comparability, 1:1 propensity score matching (PSM) was performed using a logistic regression model and the following covariates: age, sex, ASA class, surgery type, duration of procedures, hypertension, diabetes mellitus, coronary heart disease, peripheral vascular disease, preoperative Hb, preoperative β‐blockers, NSAIDs, colloids infusion, and crystalloids infusion. Matched or weighted data, Kernel density plots and standardized mean difference (SMD) were applied to assess the balance of covariates between the two groups, with an SMD <0.2 deemed as acceptable deviations for each covariate.^18^, ^19^ The association between perioperative ischemic stroke and BMI was estimated using logistic regression analysis.

Sex, coronary heart disease, peripheral vascular disease, and type of surgery were associated with the risk of high BMI complications in several previous studies.^7^, ^20^ Consequently, we conducted subgroup analyses based on these factors, using unadjusted variables with Bonferroni correction for multiple comparisons.

Statistical significance was set at *p* < 0.05. R program (version 1.4.1106, R Foundation for Statistical Computing, Vienna, Austria) and relevant packages (tableone, MatchIt, pROC, Matching, Cobalt, rms, and car) were utilized for statistical analyses.

## 3 RESULTS

### 3.1 Baseline patient characteristics

**FIGURE 2:**
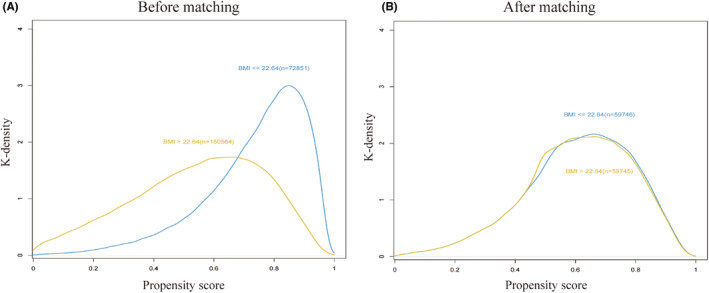
The propensity score histograms of the two groups. (A) Before matching. (B) After matching.

The study included 223,415 patients undergoing noncardiac surgery at the Chinese PLA General Hospital between January 1, 2008 and August 31, 2019. Patients were stratified into two groups based on BMI: ≤22.64 kg/m^2^ (72,851 patients) and >22.64 kg/m^2^ (150,564 patients). Table 1 provides a summary of baseline characteristics. Patients in the >22.64 kg/m^2^ group were predominantly male and older than those in the ≤22.64 kg/m^2^ group. A higher prevalence of coronary heart disease was observed in the >22.64 kg/m^2^ group, with distinct surgical preferences; joint and spinal surgeries being more common in this group, while abdominal surgeries were more prevalent in the ≤22.64 kg/m^2^ group. Propensity score matching (PSM) effectively balanced most covariates between the two BMI groups (Table 1 and Figure 2).

**TABLE 1:** Baseline characteristics unadjusted sample and propensity score‐matched sample.

Characteristic	Unadjusted sample (*N* = 223,415)			PSM adjusted (1:1) (*N* = 119,491)		
	BMI ≤22.64 (*n* = 72,851)	BMI > 22.64 (*n* = 150,564)	SMD	BMI ≤22.64 (*n* = 59,746)	BMI > 22.64 (*n* = 59,745)	SMD
Age, years	48.00 [35.00, 61.00]	53.00 [44.00, 62.00]	0.295	50.00 [37.00, 62.00]	50.00 [40.00, 59.00]	0.013
Female sex (%)	41,513 (57.0)	68,399 (45.4)	0.233	32,141 (53.8)	32,229 (53.9)	0.003
ASA physical status (%)						
Class I	32,319 (15.1)	168 (2.1)	0.158	238 (1.6)	168 (2.2)	0.119
Class II	165,421 (77.5)	5340 (66.7)		10,582 (69.8)	5250 (69.3)	
Class III	15,793 (7.4)	2500 (31.2)		4338 (28.6)	2161 (28.5)	
Class IV	693 (1.0)	1181 (0.8)		603 (1.0)	309 (0.5)	
Hypertension (%)	7688 (10.6)	36,525 (24.3)	0.368	7252 (12.1)	5637 (9.4)	0.087
Diabetes (%)	5941 (8.2)	21,702 (14.4)	0.199	5432 (9.1)	5382 (9.0)	0.003
Coronary heart disease (%)	1760 (2.4)	6424 (4.3)	0.103	1601 (2.7)	1376 (2.3)	0.024
Arterial fibrillation (%)	205 (0.3)	632 (0.4)	0.023	193 (0.3)	116 (0.2)	0.025
Valvular heart disease (%)	242 (0.3)	522 (0.3)	0.002	208 (0.3)	163 (0.3)	0.014
Myocardial infarction (%)	189 (0.3)	723 (0.5)	0.036	176 (0.3)	152 (0.3)	0.008
History of cardiac surgery (%)	140 (0.2)	356 (0.2)	0.01	130 (0.2)	92 (0.2)	0.015
History of chronic Cerebrovascular disease (%)	1706 (2.3)	5005 (3.3)	0.059	1553 (2.6)	1169 (2.0)	0.043
Stroke (%)	1227 (1.7)	3988 (2.6)	0.066	1119 (1.9)	813 (1.4)	0.041
Peripheral vascular disease (%)	2151 (3.0)	6337 (4.2)	0.068	1930 (3.2)	1627 (2.7)	0.03
Renal dysfunction (%)	752 (1.0)	1396 (0.9)	0.011	663 (1.1)	491 (0.8)	0.029
Preoperative use of β‐blockers (%)	1661 (2.3)	6852 (4.6)	0.125	1515 (2.5)	1399 (2.3)	0.013
Preoperative use of aspirin (%)	2080 (2.9)	7015 (4.7)	0.095	1810 (3.0)	1636 (2.7)	0.017
Preoperative use of statin (%)	1158 (1.6)	4420 (2.9)	0.091	1053 (1.8)	1067 (1.8)	0.002
Preoperative Hb, g/L	128.0 [117.0, 140.0]	137.0 [125.0, 149.0]	0.439	130.0 [119.0, 142.0]	131.0 [119.0, 144.0]	0.019
Preoperative ALB, g/L	41.1 [38.5, 43.6]	41.6 [39.3, 43.9]	0.161	41.2 [38.6, 43.7]	41.3 [38.9, 43.6]	0.011
Preoperative TBIL, μmol/L	10.4 [7.8, 14.4]	10.8 [8.1, 14.4]	0.048	10.6 [7.9, 14.5]	10.4 [7.8, 14.2]	0.016
Preoperative PT, s	13.2 [12.7, 13.8]	13.0 [12.5, 13.5]	0.213	13.2 [12.7, 13.7]	13.0 [12.5, 13.6]	0.129
Preoperative NLR	1.8 [1.3, 2.7]	1.8 [1.4, 2.5]	0.088	1.81 [1.34, 2.62]	1.8 [1.4, 2.6]	0.004
Preoperative PLR	122.8 [95.4, 163.7]	114.2 [90.1, 147.3]	0.192	121.1 [94.5, 160.1]	119.1 [92.9, 156.3]	0.037
Surgery type (%)						
Otolaryngology head and neck surgery	7590 (10.4)	14,248 (9.5)	0.27	6369 (10.7)	6855 (11.5)	0.05
Traumatology surgery	2244 (3.1)	4409 (2.9)		1847 (3.1)	1897 (3.2)	
Gynecological and obstetric surgery	6296 (8.6)	9122 (6.1)		4789 (8.0)	5206 (8.7)	
Abdominal surgery	21,422 (29.4)	36,219 (24.1)		16,657 (27.9)	16,030 (26.8)	
Joint surgery	3724 (5.1)	12,189 (8.1)		3186 (5.3)	2979 (5.0)	
Spinal surgery	4154 (5.7)	14,149 (9.4)		3751 (6.3)	3598 (6.0)	
Oral surgery	3744 (5.1)	5733 (3.8)		2885 (4.8)	3146 (5.3)	
Urology surgery	4690 (6.4)	13,930 (9.3)		4082 (6.8)	4121 (6.9)	
General surgery	5221 (7.2)	11,787 (7.8)		4516 (7.6)	4456 (7.5)	
Neurosurgical surgery	6419 (8.8)	13,929 (9.3)		5580 (9.3)	5378 (9.0)	
Thoracic surgery	4750 (6.5)	10,535 (7.0)		4090 (6.8)	4149 (6.9)	
Vascular surgery	614 (0.8)	1594 (1.1)		519 (0.9)	481 (0.8)	
Other	1983 (2.7)	2720 (1.8)		1475 (2.5)	1449 (2.4)	
Duration of surgery, min	147.0 [100.0, 215.0]	149.0 [102.0, 215.0]	0.005	150.0 [105.0, 219.0]	145.0 [95.0, 220.0]	0.012
Time of perioperative MAP > 60 min, min	5.0 [0.0, 15.0]	5.0 [0.0, 15.0]	0.058	5.0 [0.0, 15.0]	5.0 [0.0, 15.0]	0.037
Estimated blood loss, mL	100.0 [50.0, 200.0]	100.0 [50.0, 200.0]	0.006	100.0 [50.0, 200.0]	100.0 [50.0, 300.0]	0.164
Blood products depot						
No	63,666 (87.4)	133,701 (88.8)	0.043	52,981 (88.7)	51,255 (85.8)	0.087
Yes	9185 (12.6)	16,863 (11.2)		6765 (11.3)	8490 (14.2)	
Morphine equivalents, mg^a^	120.0 [90.0, 150.0]	123.0 [90.0, 150.0]	0.138	120.0 [90.0, 150.0]	120.0 [90.0, 150.0]	0.085
Inhalation anesthetics						
No	4696 (6.4)	8939 (5.9)	0.021	3941 (6.6)	3469 (5.8)	0.033
Yes	68,155 (93.6)	141,625 (94.1)		55,805 (93.4)	56,276 (94.2)	
Hormones						
No	13,917 (19.1)	28,878 (19.2)	0.002	11,598 (19.4)	10,057 (16.8)	0.067
Yes	58,934 (80.9)	121,686 (80.8)		48,148 (80.6)	49,688 (83.2)	
NSAIDs						
No	29,030 (39.8)	54,584 (36.3)	0.074	23,540 (39.4)	29,059 (48.6)	0.187
Yes	43,821 (60.2)	95,980 (63.7)		36,206 (60.6)	30,686 (51.4)	
Colloids infusion, mL/kg/min	10.0 [7.4, 13.5]	7.8 [5.8, 10.4]	0.567	9.3 [7.0, 12.2]	8.7 [6.2, 12.0]	0.098
Crystalloids infusion, mL/kg/min	3.2 [0.0, 5.1]	2.4 [0.0, 3.8]	0.33	3.0 [0.0, 4.7]	3.2 [1.1, 4.7]	0.084

*Note*: The data are shown as the median (interquartile range), *n* (%), or mean ± SD.

Abbreviations: ALB, albumin; ASA, American Society of Anesthesiologists; BMI, body mass index; Hb, hemoglobin; MAP, mean arterial pressure; NLR, neutrophil‐to‐lymphocyte ratio; PLR, platelet‐to‐lymphocyte ratio; PSM, propensity score matching; PT, prothrombin time; SMD, standardized mean difference; TBIL, total bilirubin.

^a^ Including those intraoperatively and postoperatively (up to 7 days after surgery). Morphine 30 mg (per os) = morphine 10 mg (iv) = sufentanil 10 μg (iv) = fentanyl 100 μg (iv) = remifentanil 100 μg (iv) = 100 mg tramadol (iv) = tramadol 200 mg (per os) = oxycodone 15 mg (per os) = dezocine 10 mg (iv) = pethidine 100 mg (iv).

### 3.2 Primary analysis

Of the entire cohort, 525 patients experienced perioperative ischemic stroke within 30 days of surgery. Unadjusted logistic regression analysis revealed a significant association between BMI >22.64 kg/m^2^ and the risk of perioperative ischemic stroke (OR 1.617; 95% CI, 1.324–1.99; *p* < 0.001; Table 2). This association persisted after adjusting for patient‐related (OR 1.283; 95% CI, 1.04–1.594; *p* < 0.05), surgery‐related (OR 1.484; 95% CI, 1.2–1.849; *p* < 0.001), and all prospectively defined confounders (OR 1.279; 95% CI, 1.025–1.607; *p* < 0.05) (Table 2). Table S3 details the complete data used in these models.

**TABLE 2:** Logistic regression and propensity score analysis of the association between high BMI and perioperative ischemic stroke.

Analysis method	OR	95% CI	*p* Value
Logistic regression analysis (*N* = 223,415)			
Model 1 (unadjusted)^a^	1.617	1.324–1.99	<0.001
Model 2 (patient‐related confounders adjusted)^b^	1.283	1.04–1.594	0.022
Model 3 (surgery‐related confounders adjusted)^c^	1.484	1.2–1.849	<0.001
Model 4 (fully adjusted)^d^	1.279	1.025–1.607	0.032
Propensity score analysis			
PS matching (*N* = 119,491)^e^	1.577	1.203–2.073	0.001

Abbreviation: CI, confidence interval; OR, odds ratio; PS, propensity score.

^a^ Model 1 was a univariable crude model.

^b^ Model 2 included age, sex, ASA Class, hypertension, diabetes mellitus, stroke, history of chronic cerebrovascular disease, coronary heart disease, arterial fibrillation, valvular heart disease, myocardial infarction, history of cardiac surgery, peripheral vascular disease, renal dysfunction, and preoperative use of β‐blockers, aspirin and statin, preoperative ALB, preoperative TBIL, preoperative PT, preoperative NLR, and preoperative PLR.

^c^ Model 3 included surgical type, duration of surgery, time of perioperative MAP >60 min, estimated blood loss, blood products depot, morphine equivalents, inhalation anesthetics, hormones, and NSAIDs, colloids infusion, and crystalloids infusion.

^d^ Model 4 includes all the above confounders. Full results are displayed in Table S3.

^e^ 119,491 pairs were matched by propensity score. Full results are displayed in Table S4.

### 3.3 PSM analysis and adjustment

PSM analysis, as described in the methods section, resulted in matched cohorts of 59,746 patients in the BMI ≤22.64 kg/m^2^ group and 59,746 patients in the BMI >22.64 kg/m^2^ group, with K‐densities similar between the two groups (Figure 2A,B). Logistic regression analysis confirmed a significant association between BMI >22.64 kg/m^2^ and the risk of perioperative ischemic stroke (OR 1.577; 95% CI, 1.203–2.073; *p* < 0.01; Table 2). Detailed data are provided in Table S4.

### 3.4 Subgroup analysis

**FIGURE 3:**
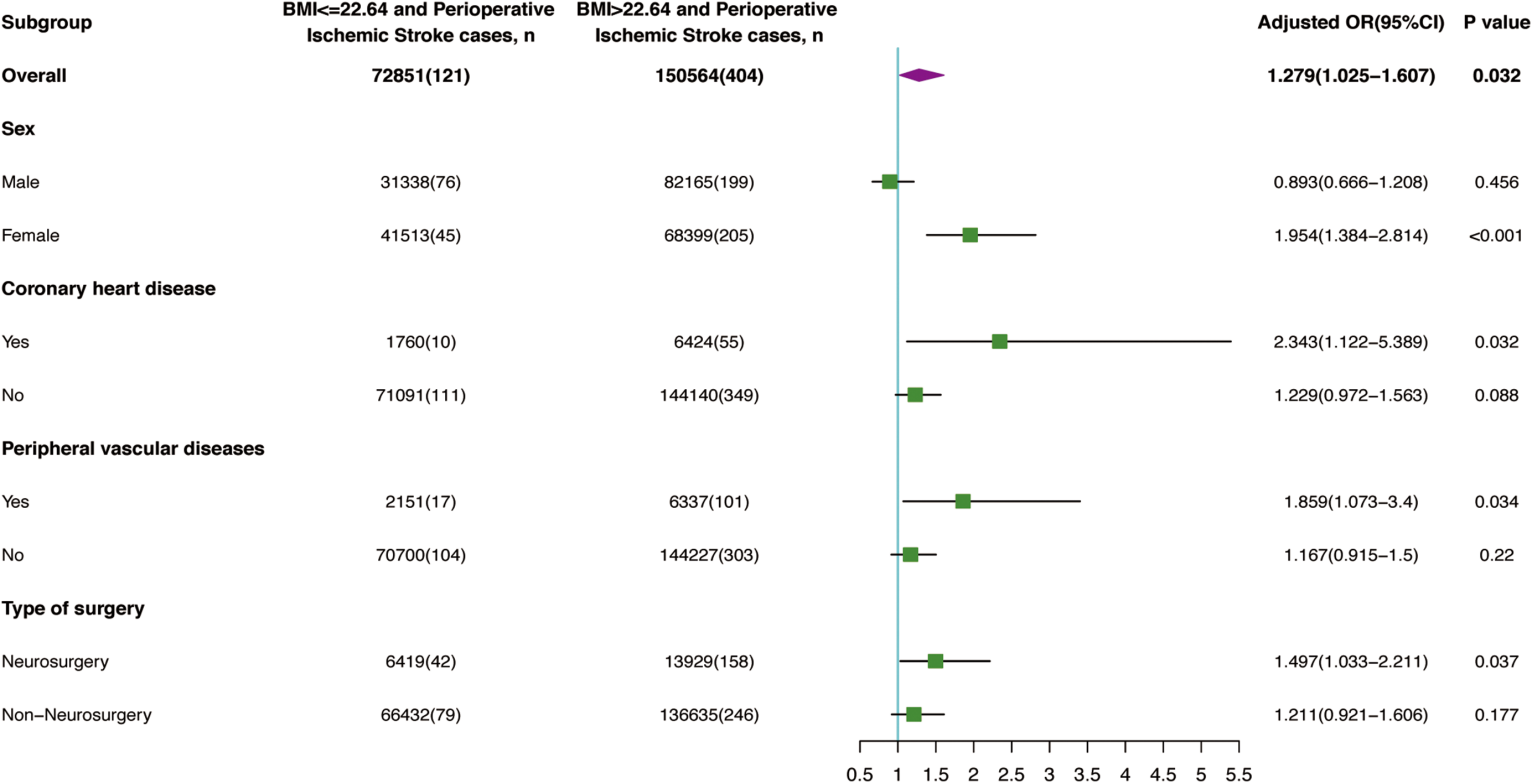
Subgroup analysis of the association between BMI and the risk of perioperative ischemic stroke. OR, odds ratio; BMI, body mass index.

Subgroup analyses based on sex, coronary heart disease, peripheral vascular disease, and surgery type were conducted (Figure 3). In the BMI >22.64 kg/m^2^ group, 41,513 (57.0%) patients were female, and the association with perioperative ischemic stroke was significant only in females (OR 1.954; 95% CI, 1.384–2.814; *p* < 0.001), but not in males (OR 0.893; 95% CI, 0.666–1.208; *p* = 0.456). Notably, BMI >22.64 kg/m^2^ was significantly associated with increased risk in patients with coronary heart disease (OR 2.343; 95% CI, 1.122–5.389; *p* = 0.032) and those with peripheral vascular disease (OR 1.859; 95% CI, 1.073–3.4; *p* = 0.034). Additionally, neurosurgery was associated with increased risk in the BMI >22.64 kg/m^2^ group (OR 1.497; 95% CI, 1.033–2.211; *p* = 0.037), while no significant association was found for non‐neurosurgery cases (OR 1.211; 95% CI, 0.921–1.606; *p* = 0.177) (Figure 3).

## 4 DISCUSSION

Perioperative stroke represents a significant and independent complication of surgery, associated with heightened risks of physical disability, cognitive dysfunction, and mortality.^21^ Recognized risk factors for perioperative stroke include hypertension, diabetes, and atrial fibrillation.^2^, ^3^, ^22^, ^23^, ^24^, ^25^, ^26^ While a BMI >30 kg/m^2^ has been established as a risk factor for stroke in general,^27^ the association between BMI and perioperative stroke remains controversial. Our study contributes to this discourse by systematically investigating the relationship between BMI as a continuous variable and the risk of perioperative ischemic stroke.

In our analysis of 223,415 patients undergoing noncardiac surgery, the overall incidence of perioperative ischemic stroke was 0.23%, aligning with international studies^28^ and a prior investigation.^29^ We innovatively explored BMI as a continuous variable to establish a clear cutoff for perioperative ischemic stroke risk. Strikingly, unadjusted data analysis demonstrated a significant association between BMI >22.64 kg/m^2^ and an increased risk of perioperative ischemic stroke. The subsequent adjustment for patient‐related and surgery‐related confounders, along with an extensive set of 34 prospectively defined confounders, consistently confirmed this association. Utilizing propensity score matching (PSM), we obtained balanced cohorts and reaffirmed a significant link between high BMI and the risk of perioperative ischemic stroke.

Our findings are notably congruent with a study involving 0.5 million Chinese individuals, where a BMI >23 kg/m^2^ was associated with an increased risk of stroke.^30^ This underscores the consistency of our results and highlights the potential clinical relevance of identifying a clear BMI threshold, such as 22.64 kg/m^2^, for preventing perioperative stroke in the Chinese surgical population. More importantly, we first identified BMI >22.64 kg/m^2^ as a substantial and independent risk factor for perioperative ischemic stroke in Chinese noncardiac surgery patients.

However, existing literature on the association between BMI and perioperative stroke has presented conflicting evidence. For example, others have identified BMI ≥25 kg/m^2^ significantly increased the risk of perioperative stroke in patients undergoing percutaneous coronary intervention,^14^ but an American study including 350,031 noncardiac, non‐neurologic patients, found BMI 35–40 kg/m^2^ appeared to have a protective effect against perioperative stroke.^3^ Varied results may be attributed to differences in surgical populations, racial disparities, and the categorical treatment of BMI, potentially overlooking the nuanced impact of normal BMI as a risk factor for perioperative stroke. Future studies with larger sample sizes and diverse populations are warranted to elucidate these relationships further.

Subgroup analyses based on sex, coronary heart disease, peripheral vascular disease, and surgery type provided additional insights. We observed a stronger association between BMI >22.64 kg/m^2^ and perioperative ischemic stroke in females, patients with coronary heart disease, those with peripheral vascular disease, and in cases involving neurosurgery. Again, this aligns with previous literature that has reported similar associations within these subgroups in separate cohorts. A previous study showed that female is associated with more than a threefold increased risk of perioperative stroke,^31^ which may be related to the slower metabolism of female patients with increased BMI having, which is more likely to cause abnormal blood lipid metabolism, resulting in the weakening of the automatic regulation of cerebral blood flow. Other studies showed that the effect of sexual dimorphism about age on the matching between local neuronal activity and regional cerebral blood flow only apparent in females but not males, it may be related to the loss of estrogen in postmenopausal women, which could lead to a greater decline in cerebral blood flow.^32^, ^33^ Similarly, others have found that patients with high BMI and diabetes have increased postoperative complications,^34^ and that the risk of stroke was higher with vascular and neurosurgical operations.^28^ Interestingly, the association of BMI > 22.64 kg/m^2^ with perioperative ischemic stroke was also evident in the neurosurgical subgroup, calling for increased attention to patients in this subgroup that must undergo neurosurgery. This subgroup analysis underscores the importance of considering patient characteristics when evaluating the impact of BMI on perioperative stroke risk.

The strengths of our study include the extensive patient sample size, the innovative exploration of BMI as a continuous variable, and the identification of a clear cutoff value for perioperative stroke risk. The integration of comprehensive preoperative, intraoperative, and perioperative data enhances the robustness of our findings. Additionally, sensitivity analyses, including PSM and subgroup analyses, consistently validated the robustness of the observed association.

Despite these strengths, our study has limitations. BMI, while commonly used, lacks precision in distinguishing lean from fat mass and providing insights into fat distribution. The single‐hospital data source may limit generalizability. Despite adjusting for numerous confounders, residual and unmeasured confounders may persist in observational studies. The risk factors causing gender differences in perioperative stroke with high BMI have not been identified. And future investigations should explore the association between high BMI and long‐term survival outcomes following perioperative ischemic stroke.

## 5 CONCLUSION

In summary, our study unequivocally establishes a significant association between BMI >22.64 kg/m^2^ and an elevated risk of perioperative ischemic stroke. BMI >22.64 kg/m^2^ emerged as an independent risk factor for perioperative ischemic stroke in our comprehensive analysis. Contrary to the conventional notion of maintaining a normal BMI as a universal standard for preventing perioperative ischemic stroke, our findings highlight the need for nuanced considerations, particularly in specific patient populations.

Our results underscore the importance of tailored perioperative weight management strategies, particularly for female patients, those with coronary heart disease and peripheral vascular disease, and individuals undergoing neurosurgery. Our study first identified BMI >22.64 kg/m^2^ as an independent risk factor that prompts a call for heightened vigilance and targeted interventions in these high‐risk subgroups to mitigate the risk of perioperative ischemic stroke. As we navigate the complexities of perioperative care, these findings contribute valuable insights to the refinement of clinical practices. Future research should explore the applicability of our results across diverse populations and delve into the long‐term implications of perioperative ischemic stroke in individuals with elevated BMI. Ultimately, our study advocates for a personalized approach to perioperative weight management to optimize patient outcomes and enhance the overall quality of surgical procedure.

## AUTHOR CONTRIBUTIONS

WM and YM conceived and designed the study. PL, RW, FL, SL, MS, and HY contributed to data extraction and acquisition. PL and RW drafted the manuscript. PL, RW, FL, ML, MQ, and LM analyzed and interpreted the data. WM and YM supervised the study. WM was the guarantor of this study, had full access to all the study data and responsible for the integrity of the data and accuracy of the data analyses. All authors contributed to the article and approved the submitted version.

## FUNDING INFORMATION

Financial support and sponsorship: This work was supported by grants from the Capital Health Research and Development of Special (2022‐4‐5025), the National Key Research and Development Program of China (No: 2018YFC2001901), and the National Natural Science Foundation of China (No. 82171464; 81801193).

## CONFLICT OF INTEREST STATEMENT

The authors have no conflict of interest to declare.

## Supporting information


Tables S1–S4.

## Data Availability

The data underlying this article will be shared on reasonable request to the corresponding author.

## References

[1] Strong K , Mathers C , Bonita R . Preventing stroke: saving lives around the world. Lancet Neurol. 2007;2:6‐187. 10.1016/S1474-4422(07)70031-5 17239805

[2] Bateman BT , Schumacher HC , Wang S , Shaefi S , Berman MF . Perioperative acute ischemic stroke in noncardiac and nonvascular surgery: incidence, risk factors, and outcomes. Anesthesiology. 2009;110(2):231‐238. 19194149 10.1097/ALN.0b013e318194b5ff

[3] George A , Mashour S , Amy M , Shanks T , Sachin S , Kheterpal S . Perioperative stroke and associated mortality after noncardiac, nonneurologic surgery. Anesthesiology. 2011;114:1289‐1296. 21478735 10.1097/ALN.0b013e318216e7f4

[4] Mashour GA , Moore LE , Lele AV , Robicsek SA , Gelb AW . Perioperative care of patients at high risk for stroke during or after non‐cardiac, non‐neurologic surgery: consensus statement from the Society for Neuroscience in anesthesiology and critical care*. J Neurosurg Anesthesiol. 2014;26(4):273‐285. 24978064 10.1097/ANA.0000000000000087

[5] Zhang F , Ma Y , Yu Y , et al. Type 2 diabetes increases risk of unfavorable survival outcome for postoperative ischemic stroke in patients who underwent non‐cardiac surgery: a retrospective cohort study. Front Aging Neurosci. 2022;13:810050. 35087397 10.3389/fnagi.2021.810050 PMC8786912

[6] Thiebaut AM , Gauberti M , Ali C , et al. The role of plasminogen activators in stroke treatment: fibrinolysis and beyond. Lancet Neurol. 2018;17(12):1121‐1132. 30507392 10.1016/S1474-4422(18)30323-5

[7] Dai H , Alsalhe TA , Chalghaf N , Riccò M , Bragazzi NL , Wu J . The global burden of disease attributable to high body mass index in 195 countries and territories, 1990–2017: an analysis of the global burden of disease study. PLoS Med. 2020;17(7):e1003198. 32722671 10.1371/journal.pmed.1003198 PMC7386577

[8] Gurm HS , Fathi R , Kapadia SR , et al. Impact of body mass index on outcome in patients undergoing carotid stenting. Am J Cardiol. 2005;96:1743‐1745. 16360369 10.1016/j.amjcard.2005.07.100

[9] Jackson RS , Sidawy AN , Amdur RL , Macsata RA . Obesity is an independent risk factor for death and cardiac complications after carotid endarterectomy. J Am Coll Surg. 2012;214(2):148‐155. 22192895 10.1016/j.jamcollsurg.2011.10.017

[10] Volkers EJ , Greving JP , Hendrikse J , et al. Body mass index and outcome after revascularization for symptomatic carotid artery stenosis. Neurology. 2017;88:2052‐2060. 28446644 10.1212/WNL.0000000000003957 PMC5440240

[11] Shah DK , Vitonis AF , Missmer SA . Association of body mass index and morbidity after abdominal, vaginal, and laparoscopic hysterectomy. Obstet Gynecol. 2015;125(3):589‐598. 25730220 10.1097/AOG.0000000000000698

[12] Chen CCG , Collins SA , Rodgers AK , Paraiso MFR , Walters MD , Barber MD . Perioperative complications in obese women vs normal‐weight women who undergo vaginal surgery. Am J Obstet Gynecol. 2007;197(1):98. 10.1016/j.ajog.2007.03.055 17618776

[13] Smith RK , Broach RB , Hedrick TL , Mahmoud NN , Paulson EC . Impact of BMI on postoperative outcomes in patients undergoing proctectomy for rectal cancer: a national surgical quality improvement program analysis. Diseases of the Colon & Rectum. 2014;57(6):687‐693. 24807592 10.1097/DCR.0000000000000097

[14] Hu YC , Yao WJ , Jin DX , et al. Bivalirudin in patients undergoing percutaneous coronary intervention and independent predictors of postoperative adverse events in these patients: a real world retrospective study. Medicine. 2021;100:e25003. 33725878 10.1097/MD.0000000000025003 PMC7969278

[15] Lee SH , Yang K , Park J , Lee JH , Lee SM . Association between high body mass index and mortality following myocardial injury after noncardiac surgery. Anesth Anal. 2020;132:960‐968. 10.1213/ANE.0000000000005303 33323785

[16] Arinze N , Farber A , Levin SR , Cheng TW , Siracuse JJ . The Association of Body Mass Index with outcomes after carotid endarterectomy. Ann Vasc Surg. 2021;77:7‐15. 34437970 10.1016/j.avsg.2021.05.046

[17] Sacco RL , Kasner SE , Broderick JP , et al. An updated definition of stroke for the 21st century a statement for healthcare professionals from the American Heart Association/American Stroke Association. Stroke. 2013;7:2064‐2089. 10.1161/STR.0b013e318296aeca PMC11078537 23652265

[18] Cheung KS , Chan EW , Chen L , Seto WK , Wong ICK , Leung WK . Diabetes increases risk of gastric cancer after helicobacter pylori eradication: a territory‐wide study with propensity score analysis. Am Diabet Assoc. 2019;42:9‐1775. 10.2337/dc19-0437 31296646

[19] Angelantonio ED , Kaptoge S , Wormser D , et al. Association of Cardiometabolic Multimorbidity with Mortality. JAMA. 2015;314:52‐60. 26151266 10.1001/jama.2015.7008 PMC4664176

[20] Guerra‐Londono CE , Han X , Penning DH . Perioperative pulmonary complications in the morbidly obese: the role of tidal volume and the type of abdominal surgery. Respir Care. 2020;65(12):1908‐1915. 32694181 10.4187/respcare.07777

[21] Philip B . Gorelick, the global burden of stroke: persistent and disabling. Lancet Neurol. 2019;18:417‐418. 30871943 10.1016/S1474-4422(19)30030-4

[22] Villareal RP , Hariharan R , Liu BC , et al. Postoperative atrial fibrillation and mortality after coronary artery bypass surgery. J Am Coll Cardiol. 2004;43(5):742‐748. 14998610 10.1016/j.jacc.2003.11.023

[23] Fallouh N , Chopra V . Statin withdrawal after major noncardiac surgery: risks, consequences, and preventative strategies. J Hosp Med. 2012;7(7):573‐579. 22744758 10.1002/jhm.1945

[24] Mcgirt MJ , Perler BA , Brooke BS , et al. 3‐Hydroxy‐3‐methylglutaryl coenzyme a reductase inhibitors reduce the risk of perioperative stroke and mortality after carotid endarterectomy. J Vasc Surg. 2005;42(5):829‐836. 16275430 10.1016/j.jvs.2005.08.039

[25] Fox CS , Golden SH , Anderson C , et al. Update on prevention of cardiovascular disease in adults with type 2 diabetes mellitus in light of recent evidence: a scientific statement from the American Heart Association and the American Diabetes Association. Diabetes Care. 2015;38(9):1777‐1803. 26246459 10.2337/dci15-0012 PMC4876675

[26] Villareal RP , Hariharan R , Liu BC , et al. Postoperative atrial fibrillation and mortality after coronary artery bypass surgery. J Am Coll Cardiol. 2004;43:742‐748. 14998610 10.1016/j.jacc.2003.11.023

[27] Strazzullo P , D'Elia L , Cairella G , Garbagnati F , Cappuccio FP , Scalfi L . Excess body weight and incidence of stroke meta‐analysis of prospective studies with 2 million participants. Stroke. 2010;41(5):e418‐e426. 20299666 10.1161/STROKEAHA.109.576967

[28] Vascular Events in Noncardiac Surgery Patients Cohort Evaluation (VISION) Study Investigators , Spence J , Lemanach Y , et al. Association between complications and death within 30 days after noncardiac surgery. Canadian Med Assoc J. 2019;191:E830‐E837. 10.1503/cmaj.190221 PMC6663503 31358597

[29] Woo SH , Marhefka GD , Cowan SW . Development and validation of a prediction model for stroke, cardiac, and mortality risk after non‐cardiac surgery. J Am Heart Assoc. 2021;10:e018013. 33522252 10.1161/JAHA.120.018013 PMC7955339

[30] Adiposity and risk of ischaemic and haemorrhagic stroke in 0·5 million Chinese men and women: a prospective cohort study. Lancet Glob Health. 2018;6(6):e630‐e640. 29773119 10.1016/S2214-109X(18)30216-X PMC5960068

[31] Hogue WC , Murphy FS , Schechtman BK , Dávila‐Román GV . Risk factors for early or delayed stroke after cardiac surgery. Circulation. 1999;100:e157‐e158. 10441102 10.1161/01.cir.100.6.642

[32] Jodie LK , Bond B , Alan RB , et al. Sex modifies the relationship between age and neurovascular coupling in healthy adults. J Cereb Blood Flow Metab. 2023;43(8):1254‐1266. 37017422 10.1177/0271678X231167753 PMC10369153

[33] Ronney BP , Aaron D , Rebecca HC , Lucy CB , Thompson GR , Jatinder SM . The effect of hypercapnia on the directional sensitivity of dynamic cerebral autoregulation and the influence of age and sex. J Cereb Blood Flow Metab. 2024;44(2):272‐283. 37747437 10.1177/0271678X231203475 PMC10993882

[34] Tang T , Tan Y , Xiao B , Zu G , Chen X . Influence of body mass index on perioperative outcomes following pancreaticoduodenectomy. Journal of Laparoendoscopic & Advanced Surgical. Dent Tech. 2020;31:999‐1005. 10.1089/lap.2020.0703 33181060

