# Supplementary material for: High body mass index is associated with elevated risk of perioperative ischemic stroke in patients who underwent noncardiac surgery: A retrospective cohort study
Source: CNS Neurosci Ther. 2024 Jul 10;30(7):e14838. doi: 10.1111/cns.14838 (PMC11236734; doi:10.1111/cns.14838)
Supplement: Supplementary file 1 — Tables S1–S4. [file CNS-30-e14838-s001.docx]

SUPPLEMENTAL MATERIAL

**Supplementary Table S1.** STROBE Statement—Checklist of Items that Should Be Included in Reports of Cohort Studies

|  | Item No | Recommendation | Page No |
| --- | --- | --- | --- |
| **Title and abstract** | 1 | (*a*) Indicate the study’s design with a commonly used term in the title or the abstract | 1 |
|  |  | (*b*) Provide in the abstract an informative and balanced summary of what was done and what was found | 2,3 |
| Introduction | | |  |
| Background/rationale | 2 | Explain the scientific background and rationale for the investigation being reported | 4,5 |
| Objectives | 3 | State specific objectives, including any prespecified hypotheses | 5 |
| Methods | | |  |
| Study design | 4 | Present key elements of study design early in the paper | 5,6 |
| Setting | 5 | Describe the setting, locations, and relevant dates, including periods of recruitment, exposure, follow-up, and data collection | 5,6 |
| Participants | 6 | (*a*) Give the eligibility criteria, and the sources and methods of selection of participants. Describe methods of follow-up | 5,6 |
|  |  | (*b*) For matched studies, give matching criteria and number of exposed and unexposed | 5,6 |
| Variables | 7 | Clearly define all outcomes, exposures, predictors, potential confounders, and effect modifiers. Give diagnostic criteria, if applicable | 6 |
| Data sources/ measurement | 8* | For each variable of interest, give sources of data and details of methods of assessment (measurement). Describe comparability of assessment methods if there is more than one group | 5,6,7 |
| Bias | 9 | Describe any efforts to address potential sources of bias | 6,7 |
| (Continued) | | | |
| **Supplementary Table S1** (Continued) | | | |
|  | Item No | Recommendation | Page No |
| Study size | 10 | Explain how the study size was arrived at | 5,6 |
| Quantitative variables | 11 | Explain how quantitative variables were handled in the analyses. If applicable, describe which groupings were chosen and why | 6,7 |
| Statistical methods | 12 | (*a*) Describe all statistical methods, including those used to control for confounding | 6,7 |
|  |  | (*b*) Describe any methods used to examine subgroups and interactions | 7 |
|  |  | (*c*) Explain how missing data were addressed | Not applicable |
|  |  | (*d*) If applicable, explain how loss to follow-up was addressed | Not applicable |
|  |  | (*e*) Describe any sensitivity analyses | 7 |
| Results | | |  |
| Participants | 13* | (a) Report numbers of individuals at each stage of study—eg numbers potentially eligible, examined for eligibility, confirmed eligible, included in the study, completing follow-up, and analysed | 5,6 |
|  |  | (b) Give reasons for non-participation at each stage | Not applicable |
|  |  | (c) Consider use of a flow diagram | Figure 1 |
| Descriptive data | 14* | (a) Give characteristics of study participants (eg demographic, clinical, social) and information on exposures and potential confounders | Table 1 |
|  |  | (b) Indicate number of participants with missing data for each variable of interest | Not applicable |
|  |  | (c) Summarise follow-up time (eg, average and total amount) | Not applicable |
| Outcome data | 15* | Report numbers of outcome events or summary measures over time | 5 |
| (Continued) | | | |
| **Supplementary Table S1** (Continued) | | | |
|  | Item No | Recommendation | Page No |
| Main results | 16 | (*a*) Give unadjusted estimates and, if applicable, confounder-adjusted estimates and their precision (eg, 95% confidence interval). Make clear which confounders were adjusted for and why they were included | 7,8  Table 2, Supplementary Table S3, Supplementary Table S4 |
|  |  | (*b*) Report category boundaries when continuous variables were categorized | 7,8 |
|  |  | (*c*) If relevant, consider translating estimates of relative risk into absolute risk for a meaningful time period | Not applicable |
| Other analyses | 17 | Report other analyses done—eg analyses of subgroups and interactions, and sensitivity analyses | 8 |
| Discussion | | |  |
| Key results | 18 | Summarise key results with reference to study objectives | 9,10 |
| Limitations | 19 | Discuss limitations of the study, taking into account sources of potential bias or imprecision. Discuss both direction and magnitude of any potential bias | 11 |
| Interpretation | 20 | Give a cautious overall interpretation of results considering objectives, limitations, multiplicity of analyses, results from similar studies, and other relevant evidence | 9,10,11 |
| Generalisability | 21 | Discuss the generalisability (external validity) of the study results | Not applicable |
| Other information | | |  |
| Funding | 22 | Give the source of funding and the role of the funders for the present study and, if applicable, for the original study on which the present article is based | 13 |

**Supplementary Table S2.** ICD-9/10 Diagnosis Codes for Ischemic Stroke

| Ischemic stroke | ICD-9/ICD-10 | 433.X1/I63.X | Occlusion and stenosis of precerebral arteries with cerebral infarction |
| --- | --- | --- | --- |
|  | ICD-9 | 434.X1 | Occlusion of cerebral arteries with cerebral infarction |
|  | ICD-9/ICD-10 | 437.1/I67.81,  I67.89 | Other generalized ischemic cerebrovascular disease |
|  | ICD-9/ICD-10 | 437.9/I67.9 | Unspecified cerebrovascular disease |

**Supplementary Table S3**. Univariate and Multivariate Logistic Regression Analysis for Perioperative Stroke in Model 4.

| **Variables** | **Logistic regression analysis** | | | |  |  |
| --- | --- | --- | --- | --- | --- | --- |
|  | **OR** | **CI** | | **P** |  |  |
| **BMI>22.64** | 1.279225305 | 1.025-1.607 | | 0.0318 |  |  |
| **Age** | 1.044039299 | 1.035-1.053 | | 8.32E-23 |  |  |
| **Sex ( female)** | 1.207276726 | 0.981-1.485 | | 0.0748 |  |  |
| **ASA physical status (%)** |  |  | |  |  |  |
| Class II | 1.233257385 | 0.777-2.086 | | 0.4027 |  |  |
| Class III | 1.583328076 | 0.954-2.77 | | 0.0892 |  |  |
| Class IV | 2.456883695 | 1.269-4.876 | | 0.0086 |  |  |
| **Myocardial infarction** | 1.288250421 | 0.617-2.409 | | 0.4618 |  |  |
| **History of cardiac surgery** | 1.497545548 | 0.554-3.384 | | 0.3745 |  |  |
| **Cerebrovascular disease** | 7.119319227 | 4.985-10.072 | | 6.86E-28 |  |  |
| **Hypertension** | 1.391818048 | 1.134-1.709 | | 0.0016 |  |  |
| **Diabetes mellitus** | 1.482365223 | 1.205-1.816 | | 0.0002 |  |  |
| **Stroke** | 0.911269074 | 0.639-1.309 | | 0.6115 |  |  |
| **Coronary heart disease** | 0.967118344 | 0.705-1.306 | | 0.8314 |  |  |
| **Renal dysfunction** | 1.381477105 | 0.73-2.397 | | 0.2831 |  |  |
| **Valvular heart disease** | 2.436555472 | 0.996-5.066 | | 0.0297 |  |  |
| **Arterial fibrillation** | 1.396688159 | 0.721-2.49 | | 0.2877 |  |  |
| **Peripheral vascular disease** | 1.093819565 | 0.834-1.425 | | 0.5110 |  |  |
| **Preoperative Hb** | 1.002871658 | 0.997-1.009 | | 0.3699 |  |  |
| **Preoperative TBIL** | 0.999590232 | 0.996-1.002 | | 0.7984 |  |  |
| **Preoperative ALB** | 0.961601595 | 0.938-0.986 | | 0.0021 |  |  |
| **Preoperative PT** | 1.031213632 | 0.974-1.068 | | 0.1631 |  |  |
| **Preoperative use of β-blockers** | 1.320647823 | 0.982-1.754 | | 0.0602 |  |  |
| **Preoperative use of aspirin** | 3.691099303 | 2.883-4.712 | | 1.90E-25 |  |  |
| **Preoperative NLR** | 1.015621927 | 0.997-1.033 | | 0.0823 |  |  |
| **Preoperative PLR** | 1.000788362 | 1-1.002 | | 0.0818 |  |  |
| **Preoperative use of statin** | 0.641421316 | 0.458-0.885 | | 0.0081 |  |  |
| **Surgery type** |  |  | |  |  |  |
| Trauma surgery | 0.162353034 | 0.06-0.369 | | 0.0081 |  |  |
| Gynecology and obstetrics | 0.341690639 | 0.167-0.653 | | 6.36E-05 |  |  |
| Abdominal surgery | 0.245280306 | 0.16-0.381 | | 0.0019 |  |  |
| Joint surgery | 0.483219253 | 0.3-0.783 | | 1.87E-10 |  |  |
| (Continued) | | | | |  | 0.777-2.086 |
| **Supplementary Table S3** (Continued) | | | | |  |  |
| **Variables** | **Logistic regression analysis** | | | |  |  |
|  | **OR** | **CI** | **P** | |  |  |
| Spinal surgery | 0.670736285 | 0.435-1.046 | | 0.0734 |  |  |
| Oral surgery | 0.61453739 | 0.333-1.089 | | 0.1045 |  |  |
| Urology surgery | 0.287052997 | 0.166-0.488 | | 5.09E-06 |  |  |
| General surgery | 0.136780228 | 0.041-0.344 | | 0.0002 |  |  |
| Vascular surgery | 0.308962488 | 0.159-0.582 | | 0.0004 |  |  |
| Neurosurgery | 1.631212343 | 1.111-2.445 | | 0.0147 |  |  |
| Chest surgery | 0.261210228 | 0.14-0.468 | | 1.10E-05 |  |  |
| Others | 0.348101773 | 0.103-0.89 | | 0.0496 |  |  |
| **Duration, min** | 1.001768061 | 1.001-1.003 | | 0.0005 |  |  |
| **Blood loss** | 1.000012819 | 1.000-1.000 | | 0.8739 |  |  |
| **MAP>60, min** | 1.120915852 | 0.925-1.355 | | 0.2414 |  |  |
| **Morphine equivalents** | 1.000648158 | 0.999-1.003 | | 0.5389 |  |  |
| **Blood products depot** | 1.120290561 | 0.847-1.474 | | 0.4216 |  |  |
| **Inhalation anesthetics** | 0.927225474 | 0.617-1.46 | | 0.7297 |  |  |
| **Hormones** | 1.176350606 | 0.926-1.513 | | 0.1945 |  |  |
| **NSAIDs** | 1.249206687 | 0.994-1.576 | | 0.0583 |  |  |
| **Crystalloids infusion** | 0.993624672 | 0.966-1.021 | | 0.6469 |  |  |
| **Colloids infusion** | 1.066147645 | 1.024-1.109 | | 0.0016 |  |  |

| Abbreviations: BMI, body mass index; ASA, American Society of Anesthesiologists; Hb, hemoglobin; ALB, albumin; TBIL, total bilirubin; NLR, neutrophil-lymphocyte ratio; PLR, platelet to lymphocyte ratio; PT, prothrombin time; PF, plasma fibrinogen; MAP, mean arterial pressure; NSAIDs, nonsteroidal anti-inflammatory drugs. |
| --- |

**Supplementary Table S4**. Univariate Logistic Regression Analysis for Perioperative Stroke in the PS Matched Cohort.

| **Variables** | **Logistic regression analysis** | | |
| --- | --- | --- | --- |
|  | **OR** | **CI** | **P** |
| **BMI>22.64** | 1.577 | 1.203-2.073 | 0.001 |
| **Age** | 1.049 | 1.036-1.061 | 2.71596E-15 |
| **Sex ( female)** | 0.987 | 0.731-1.331 | 0.9304 |
| **ASA physical status (%)** |  |  |  |
| Class II | 1.402 | 0.806-2.651 | 0.2623 |
| Class III | 1.524 | 0.807-3.067 | 0.2129 |
| Class IV | 2.393 | 0.909-6.281 | 0.0754 |
| **Myocardial infarction** | 0.526 | 0.082-1.862 | 0.3966 |
| **History of cardiac surgery** | 0.558 | 0.029-3.16 | 0.5907 |
| **Cerebrovascular disease** | 5.845 | 3.314-10.037 | 3.87177E-10 |
| **Hypertension** | 1.044 | 0.745-1.45 | 0.7985 |
| **Diabetes mellitus** | 1.633 | 1.177-2.234 | 0.0027 |
| **Stroke** | 1.472 | 0.836-2.645 | 0.1871 |
| **Coronary heart disease** | 0.906 | 0.517-1.508 | 0.7156 |
| **Renal dysfunction** | 1.994 | 0.844-4.115 | 0.0844 |
| **Valvular heart disease** | 2.867 | 0.635-8.789 | 0.1067 |
| **Arterial fibrillation** | 1.652 | 0.612-3.853 | 0.2803 |
| **Peripheral vascular disease** | 0.965 | 0.611-1.492 | 0.8750 |
| **Preoperative Hb** | 1 | 0.991-1.009 | 0.9380 |
| **Preoperative TBIL** | 1 | 0.996-1.004 | 0.7927 |
| **Preoperative ALB** | 0.976 | 0.941-1.013 | 0.1951 |
| **Preoperative PT** | 1.046 | 0.964-1.1 | 0.1678 |
| **Preoperative use of β-blockers** | 1.36 | 0.796-2.223 | 0.2390 |
| **Preoperative use of aspirin** | 4.778 | 3.24-6.99 | 1.45639E-15 |
| **Preoperative NLR** | 1.011 | 0.986-1.032 | 0.3542 |
| **Preoperative PLR** | 1.001 | 1-1.002 | 0.0090 |
| **Preoperative use of statin** | 0.448 | 0.24-0.788 | 0.0078 |
| **Surgery type** |  |  |  |
| Trauma surgery | 0.149 | 0.048-0.378 | 0.0002 |
| Gynecology and obstetrics | 0.216 | 0.071-0.536 | 0.0024 |
| Abdominal surgery | 0.157 | 0.09-0.276 | 8.56643E-11 |
| Joint surgery | 0.304 | 0.144-0.614 | 0.0012 |
| (Continued) | | | |

| **Supplementary Table S4** (Continued) | | | | |
| --- | --- | --- | --- | --- |
| **Variables** | **Logistic regression analysis** | | | |
|  | **OR** | **CI** | **P** | |
| Spinal surgery | 0.58 | 0.315-1.053 | | 0.0755 |
| Oral surgery | 0.59 | 0.289-1.141 | | 0.1282 |
| Urology surgery | 0.207 | 0.089-0.438 | | 8.64209E-05 |
| General surgery | 0.052 | 0.003-0.247 | | 0.0038 |
| Vascular surgery | 0.18 | 0.065-0.449 | | 0.0005 |
| Neurosurgery | 1.43 | 0.88-2.368 | | 0.1554 |
| Chest surgery | 0.191 | 0.082-0.409 | | 4.59935E-05 |
| Others | 0.306 | 0.071-0.906 | | 0.060 |
| **Duration, min** | 1.001 | 0.999-1.002 | | 0.4087 |
| **Blood loss** | 1 | 1-1 | | 0.8534 |
| **MAP>60, min** | 1.243 | 0.936-1.643 | | 0.1292 |
| **Morphine equivalents** | 1 | 0.997-1.003 | | 0.8904 |
| **Blood products depot** | 1.312 | 0.882-1.935 | | 0.1753 |
| **Inhalation anesthetics** | 0.801 | 0.462-1.515 | | 0.4604 |
| **Hormones** | 1.344 | 0.921-2.027 | | 0.1410 |
| **NSAIDs** | 1.439 | 1.044-1.991 | | 0.0270 |
| **Crystalloids infusion** | 0.991 | 0.951-1.031 | | 0.6522 |
| **Colloids infusion** | 1.045 | 0.99-1.102 | | 0.1013 |

| Abbreviations: BMI, body mass index; ASA, American Society of Anesthesiologists; Hb, hemoglobin; ALB, albumin; TBIL, total bilirubin; NLR, neutrophil-lymphocyte ratio; PLR, platelet to lymphocyte ratio; PT, prothrombin time; PF, plasma fibrinogen; MAP, mean arterial pressure; NSAIDs, nonsteroidal anti-inflammatory drugs. |
| --- |
